# Research on slurry diffusion and seepage law in mining overburden fractures based on CFD numerical method

**DOI:** 10.1038/s41598-023-48828-5

**Published:** 2023-12-02

**Authors:** Cao Zhengzheng, Jia Yunlong, Li Zhenhua, Du Feng

**Affiliations:** 1 International Joint Research Laboratory of Henan Province for Underground Space Development and Disaster Prevention, School of Civil Engineering, Henan Polytechnic University, Jiaozuo, 454000 Henan China; 2 Henan Mine Water Disaster Prevention and Control and Water Resources Utilization Engineering Technology Research Center, Henan Polytechnic University, Jiaozuo, 454000 Henan China; 3 Collaborative Innovation Center of Coal Work Safety and Clean High Efficiency Utilization, Jiaozuo, 454000 Henan China

**Keywords:** Coal, Civil engineering

## Abstract

It is of great theoretical significance and engineering application value to research the diffusion law of slurry in mining fractures of rock strata to enrich grouting theory and improve grouting sealing effect. In this paper, the law of grout diffusion in fractures under different working conditions is systematically explored and analyzed, and a numerical simulation scheme of grout diffusion in a single slab crack is established. Then, the diffusion law of grouting slurry in crack under different rheological index and different consistency index is further investigated. The results show that the diffusion time of grouting slurry has no relation with the rheological index. The grout pressure at the same point increases with the increase of rheological index. When the rheological index increases by 0.1, the grout pressure increases by about 12.5%. The closer the grouting mouth is, the more the grouting pressure is affected by the rheological index. There is little relationship between the diffusion time of grouting slurry and consistency index. The grout pressure at each measurement point increases with the increase of the consistency index. When the consistency index increases by 1, the grout pressure increases by about 15% on the basis of the origin. The closer the grouting mouth is, the more the grouting pressure is affected by the consistency index. In engineering practice, when grouting slurry with large rheological index or consistency index exists, it is necessary to moderately increase the grouting pressure value.

## Introduction

The environment of coal mine construction site is complex and changeable, and there are a lot of goaf water and harmful gases such as carbon monoxide and gas left in the goaf. In addition, mining stress has led to the movement and destruction of overlying rock, forming a large range of water-conducting fracture zones. Mining work of lower carboniferous coal seams causes secondary damage of overlying rock, and induces the development of mining fracture again. Finally, the water-conducting fracture zone is redistributed, which is highly likely to lead to the connection of double-series coal seams and the discharge of goaf water and harmful gases, which poses a serious threat to the coal miners^1^. Therefore, it is of great significance for the healthy development of coal mining industry to study the proportion of grouting slurry, establish a reasonable grouting method, and improve the grouting level in coal mine^2,3^.

Many scholars have studied the diffusion law of grouting slurry in mining fractures. Yan et al.^4,5^ pointed out that computer aided optimization of cement-based materials proportion can accurately quantify the accompanying microstructure evolution, material composition, and the mechanical properties changes. Wang et al.^6^ built a physical model of mining up and down the test mine through field measurement, similar simulation and theoretical analysis, and analyzed the dynamic evolution and mechanism of water inrush in karst roof under different mining sequences. Wei et al.^7^ identified the relationship between thixotropy mechanism and macroscopic thixotropy intensity by analyzing the changes of microstructure and pores in loess during thixotropy. Wang et al.^8–10^ studied the changes of fracture surface morphology and surface roughness parameters caused by cooling treatment. Xiaolei et al.^11^ pointed out that when the collapse column of overburden is disturbed by the working face, the grain loss in the karst collapse column occurs by the dissolution and corrosion of groundwater, thereby inducing the water inrush disaster. Peiding et al.^12^ the temporal and spatial evolution mechanism of the water-conducting fractured zone of overlying strata in the Kongzhuang coal mine is revealed, which provides the theoretical guidance for the prediction and prevention of water inrush disaster in the coal mine with the similar mining conditions. Tao et al.^13^ studied the force and flow characteristics of inorganic solidified foam slurry in the gas leakage crack, and compared and verified the rationality of the theory and numerical model of horizontal crack diffusion at constant air leakage rate. Yinshan^14^ studied the influence of grouting separated strata zone on water retention in formation through numerical simulation, and concluded that the higher the height of grouting backfilling is, the better effect the water retention is. Bin et al.^15^ derived the diffusion radius equation of Bingham fluid grout in rough inclined cracks on the basis of considering the pulp-water separation characteristics of grout, which mainly included four factors, containing crack width, crack roughness, crack inclination angle and grouting pressure difference. Maolin et al.^16^ studied the influence of particle size of coal dust on the wetting ability of surfactant mixed solution, and concluded that particle size of coal dust does not affect the synergistic wetting effect between surfactant, but affect the synergistic wetting degree between surfactant. Yuhao^17^ studied the migration rules of grout with different water-cement ratios and the denaturing characteristics of mudstone during the grouting process of fractured mudstone, and carried out multiple sets of seepage tests with single fracture and multi-fractures, and obtained the influence rules of grout water-cement ratio and fracture roughness on seepage characteristics of fractured mudstone under pressure conditions. Dongliang et al.^18^ studied the diffusion law of Newtonian fluid grout in cracks, and concluded that the diffusion range of Newtonian fluid grout is negatively correlated with crack opening and grout viscosity, and positively correlates with grout flow rate. The grout pressure loss is proportional to the grout flow rate and the grout viscosity, and inversely proportional to the third square of the crack opening. Jinxi et al.^19^ studied the influence of different water-cement ratio, grouting pressure and grouting time on the grout diffusion process of fractured rock mass with three-dimensional network, and believed that the pressure should be gradually increased during grouting to ensure that the grout fully penetrates into the cracks. Heyan et al.^20^ studied the effect of temperature on the diffusion behavior of self-expanding polymer grout in cracks. The study showed that under the same grouting volume, the higher the preheating temperature is, the faster the diffusion rate of grout and the earlier the initial setting time is. Tianqi et al.^21^ studied the scouring effect of moving water on the grout in the process of moving water grouting. It is necessary to use water to scour the grout mainly along both sides of the diffusion body, so that part of the grout is separated from the main body and the grout is lost. Hongbo et al.^22^ studied the diffusion law of grout in horizontal hole grouting of fractured rock mass, and the results showed that the gravity has a great influence on the diffusion form, diffusion distance and final grouting pressure of grout in horizontal hole grouting, and the influence of gravity on the diffusion law of grout in horizontal hole grouting cannot be ignored. Zhaoxing et al.^23^ studied the diffusion law of grouting slurry in horizontal hole of inclined fracture, and the results showed that under the same conditions, the diffusion distance of grouting slurry in the crack above the horizontal grouting hole is larger than that in the crack below the horizontal grouting hole, and the gap presents a multi-fold increase under the scale of wide tensile crack. Yang et al.^24^ studied the law of grout diffusion in rough cracks of rock mass, and the results showed that the grout pressure difference increases with the increase of grout dynamic viscosity, and the greater the grout viscosity is, the more significant the influence of crack roughness on grout pressure is. Zhuo et al.^25^ studied the diffusion law of a single fissure slurry under the action of moving water. With the increase of diffusion distance, the slurry gradually shows uneven diffusion in different directions. Along the direction of diffusion against water, the curve of diffusion range converges rapidly to the limit diffusion range. Along the water diffusion direction, the diffusion range continues to increase with time, and the slurry gradually tends to spread at an equal rate.

Through high pressure grouting, the grouting slurry fills and blocks the fracture channels in the rock formation, forming the construction of water and gas-resisting key strata. The fractures caused by mining stress destroy the water storage function of overlying aquifer and cannot be repaired by itself. It is obvious that the research achievements of slurry diffusion and seepage law have been obtained by laboratory experiment, numerical simulation, and theoretical analysis. However, the slurry diffusion and seepage law in mining overburden fractures based on CFD numerical method has not been carried out at present. The way of artificial grouting to seal the water-conducting fracture zone can block the aquifer leakage channel to the goaf, so that the aquifer damaged by mining can regain the water storage function and realize the artificial restoration of the aquifer^26^. On this basis, the paper takes the single slab crack as the research object and use the Herschel-Bulkley mathematical model to study the grout diffusion. The theoretical and numerical models of slurry flow diffusion are established to analyze diffusion characteristics and pressure field distribution characteristics of grouting slurry in a single slab crack, which provides the theoretical references for the design, construction and numerical modeling of underground grouting engineering practice in coal mine.

## The theoretical principle of CFD numerical method

### Basic assumptions

The basic assumptions are put forward to build a mathematical model of grouting diffusion in a single slab crack.

1. The slurry is an isotropic and in-compressible homogeneous fluid. During the whole grouting process, the slurry flow pattern remains unchanged and the heat term is ignored.
2. The flow of slurry in cracks belongs to laminar flow, and the flow process conforms to the continuous differential equation.
3. The slurry flow process is not affected by external vibration and groundwater.
4. The single horizontal crack meets the boundary condition of no slip.
5. The slurry flow process does not penetrate, and does not chemically react with the rock formation.

### Mathematical model of grouting slurry

**Figure 1:**
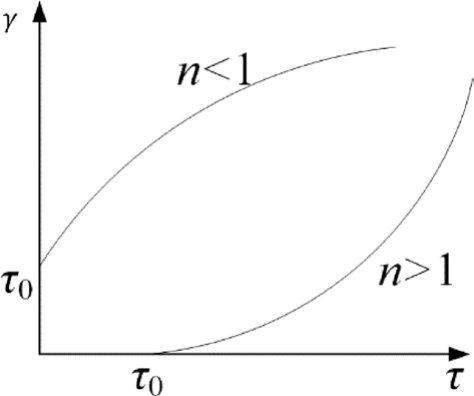
Herschel-Bulkley fluid flow curve.

The Herschel-Bulkley mathematical model, simplified as the H-B model, is a parabola with intercepts on the axis, shown in Fig. 1, and the intercept value is the yield stress of a Bingham fluid. The rheological equation for a Bingham fluid is as follows,

1 $$\tau = \tau_{0} + K\gamma^{n}$$

where $\tau$ is the shear stress of the slurry; $\tau_{0}$ is the yield stress threshold; $K$ is the consistency index; $n$ is the power-law index; $\mu_{0}$ is yield viscosity.

After making appropriate assignments for each parameter, the H-B model can simulate Bingham fluids and power-law fluids respectively. It is widely employed and suitable for a variety of scenarios, therefore the Herschel-Bulkley model is introduced to simulate the slurry.

### Numerical calculation software

In this paper, the Fluent software in Ansys, a numerical model software, is used for numerical simulation calculation of slurry diffusion test. Fluent is a widely popular kind of CFD software at home and abroad, which contains rich physical models, relatively advanced numerical methods and powerful post-processing functions, which is suitable for the research work in this article. More precisely, Fluent software adopts a finite-volume method based on completely unstructured grid, and has gradient algorithm based on grid nodes and grid units. Besides, Fluent can achieve optimum convergence speed and precision, by adopting a variety of solutions and multiple grid acceleration convergence techniques. The finite volume method (FVM) is mainly used for calculation. The VOF model can simulate two or more fluids that cannot be mixed with each other by solving a separate momentum equation and processing the volume fraction of each fluid that passes through the region, namely, it is suitable for independent calculation of the volume fraction of each phase of a multi-fluid in a flow field with a clear phase interface^27^. In the grouting test studied in this paper, grouting slurry and gas are immiscible and have a clear interface. Therefore, the VOF model is employed to obtain arbitrary interface in steady state or instantaneous state by solving the volume rate occupied by two fluid components, so as to study the flow state of grouting slurry in a single crack.

## Correctness verification of grouting numerical model of smooth single crack

### Basic steps of numerical simulation of smooth slab single crack

(1)(1) Establishment of smooth single crack model

**Figure 2:**
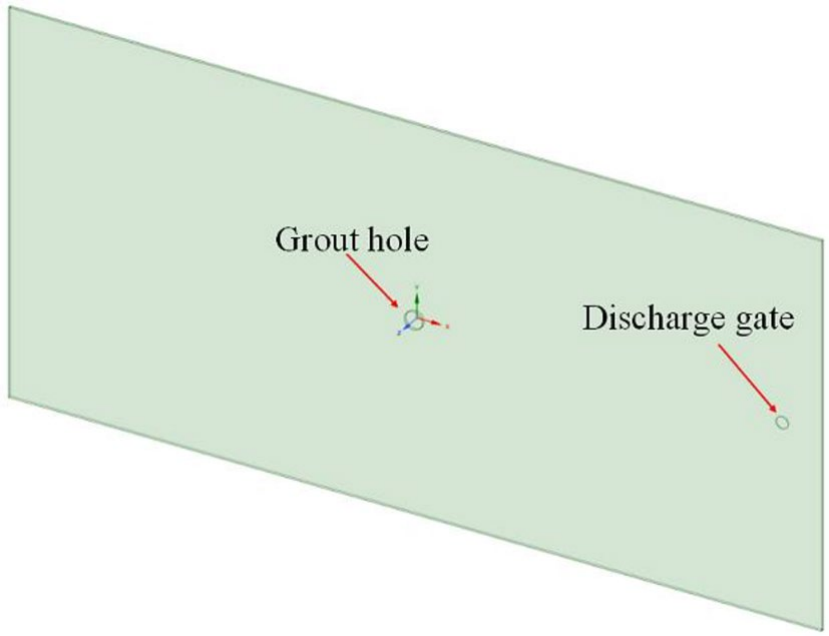
Single slab fracture model.

**Figure 3:**
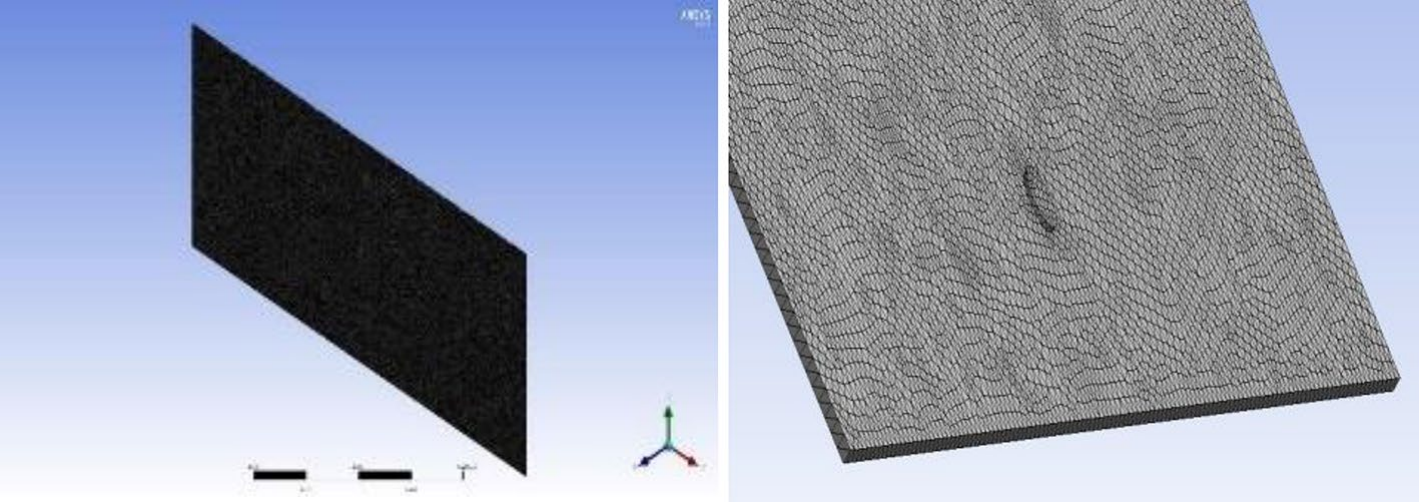
Grid diagram of a single slab fracture.

Spaceclaim in Ansys software is used to build the fracture model. The fracture model is consistent with the grouting equipment in laboratory test, and the fracture size is 1350 × 650 × 5mm (length × width × height). Workbench's mesh module is used for meshing, the mesh size is 5mm, and the grouting port is partially encrypted. After the meshing is completed, the mesh quality is checked in the Quality window, and the quality of most meshes is close to 1. The single flat fracture model and meshing are shown in Figs. 2 and 3.

(2)(2) Smooth single crack solution setup and simulation scheme.

The settings in Fluent solver are shown in Table 1.

**Table 1:** Solver parameter configuration.

Items in solver settings	The parameter value
Physical model	VOF
Time item	Transient
Sticky model	laminar flow
Number of fluid phases	Liquid phase (slurry), gas phase (gas)
Serous rheological model	Herschel-Bulkley model
Boundary conditions	Speed inlet

**Figure 4:**
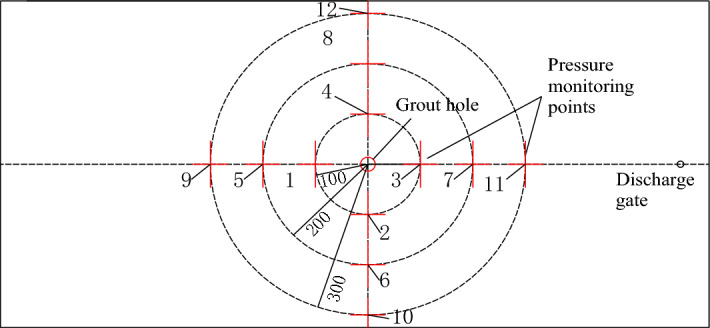
Layout of measuring points of grouting fluid diffusion in a single slab crack.

The slurry and gas parameters are shown in Tables 2 and 3, respectively (Fig. 4).

**Table 2:** Gas parameter.

Density (kg/m^3^)	Viscosity (kg/m)
1.225	1.7894 × 10^–5^

**Table 3:** Grouting slurry parameter.

Rheological model	H-B
Density *ρ* (Kg/m^3^)	1250
Yield stress $\tau_{0}$(Pa)	10.37
Consistency index *K*	1.51
Power-law exponent *n*	0.89

H-B rheological model is used to conduct numerical simulation calculation on the diffusion of grout in a single slab crack. This numerical simulation test studies the diffusion law of grout in the radial stage, and the arrangement of each pressure measurement point is the same as that of the seepage test of grout in the visual single crack, shown in Fig. 7. Therefore, grouting is stopped when grouting slurry diffuses to the critical surface of the radial diffusion stage and the bidirectional diffusion stage. The numerical simulation scheme of grouting slurry is shown in Table 4.

**Table 4:** Numerical simulation scheme of grouting fluid diffusion in single slab crack (Grouting speed).

Number of groups	Grouting speed *v* (L/h)	Roughness *R*	Fracture opening *h* (mm)
1	2.1	0	5
2	3.25	0	5
3	4.2	0	5
4	5.25	0	5
5	6.3	0	5

### Result analysis of grouting diffusion law in single plate crack

**Figure 5:**
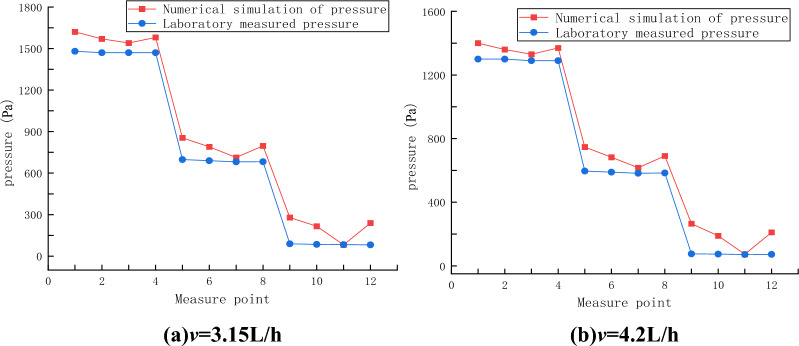
Change curve of slurry pressure.

After the completion of the numerical simulation test of the above test groups, the test data such as slurry pressures are extracted by the post-processing software CFD-Post, and the data is processed by the origin software, and the corresponding curve is drawn. For example, the slurry pressure curve data of measurement points are extracted and plotted as a curve plot for further analysis, and the slurry pressure change curves of measurement points in 2# and 3# test groups of the C-class are shown in Fig. 5.

**Figure 6:**
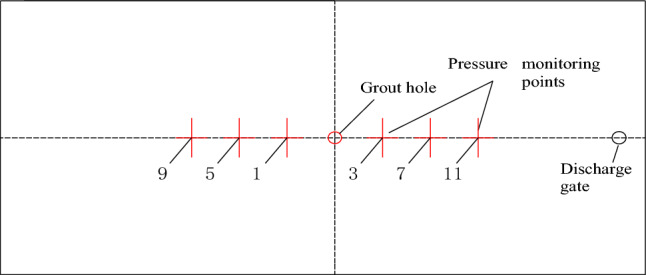
Layout of measurement points T_1_, T_3_, T_5_, T_7_, T_9_, T_11_.

The pressure change curves of laboratory test and numerical simulation with time when the grouting speed is *v* = 3.15L/h and *v* = 4.2L/h are shown in Fig. 5. it is obvious that the curve changes are similar in the comparison test of two experimental groups. In the laboratory test, the slurry pressure values of four measurement points at the same distance from the grouting hole are basically the same, and the pressure values of each measurement point are small. In the numerical simulation test, the pressure value of each measurement point is greater than that of the corresponding laboratory test. In addition, at the four measurement points located on the circumference 100 mm away from the grouting hole, the pressure value of measuring point T_1_ is the largest, the pressure value of T_3_ is the smallest, and the pressure value of T_2_ and T_4_ is relatively close. This law also exists on the circumference of 200 mm and 300 mm of the grouting hole, namely, the pressure values of T_5_ and T_9_ are larger, and the pressure values of T_7_ and T_11_ are smaller. The pressure values of T_6_ and T_8_, T_10_ and T_12_ are similar. After in-depth analysis, it is concluded that:

1. Numerical simulation result is more ideal than that of laboratory experiment. Numerical simulation is more idealized than laboratory test. In the process of laboratory test, due to the limitation of test conditions, complete seal cannot be achieved in diffusion test. However, numerical simulation is different, and complete idealized seal can be achieved by setting boundary conditions to maintain perfect gas tightness. Therefore, the pressure measured in the single crack seepage test is low, and it is greatly affected by the gas pressure. However, the gas tightness of the corresponding single crack in numerical simulation is controlled by the boundary conditions. Compared with the laboratory test, the gas tightness is better, so the measured pressure value is larger under the same conditions.
2. The numerical model of single-crack seepage flow is shown in Fig. 4. As mentioned above, the single-crack numerical model is almost completely closed, and only the grouting hole and discharge port communicate with the outside atmosphere. The grouting hole is used to inject slurry, which do not communicate with the outside during the test, so only the discharge port can adjust the atmospheric pressure in the single crack. The discharge port is set on the left side of the single crack, namely, closer to the measurement points T_3_, T_7_ and T_11_, resulting in lower pressure values of these three measurement points compared with other measurement points on the same circumference. Similarly, the pressure values of three measurement points T_1_, T_5_ and T_9_ on the left side of the single crack are the largest. The other measurement points are symmetrically distributed on the single crack, and the distance from the discharge port is centered, so the pressure value of the other measurement points is relatively close and centered.
3. The pressure values of four measurement points at the same distance from the grouting hole are basically the same in the early stage of grouting test, while the difference in pressure values occurred in the late stage of grouting test, shown in Fig. 6. According to the analysis, in the early stage of grouting test, there is no obvious difference in the pressure values at the left and right measurement points because the grouting slurry does not fill the cracks and does not completely seal the space on the left side of the cracks. However, in the late stage of grouting test, the grouting slurry almost fills the entire crack in the direction of the crack height, thus forming a good seal on the left space. At this time, the pressure value of the left and right side measurement points begins to show a gap, and the gap is getting wider and wider.

**Figure 7:**
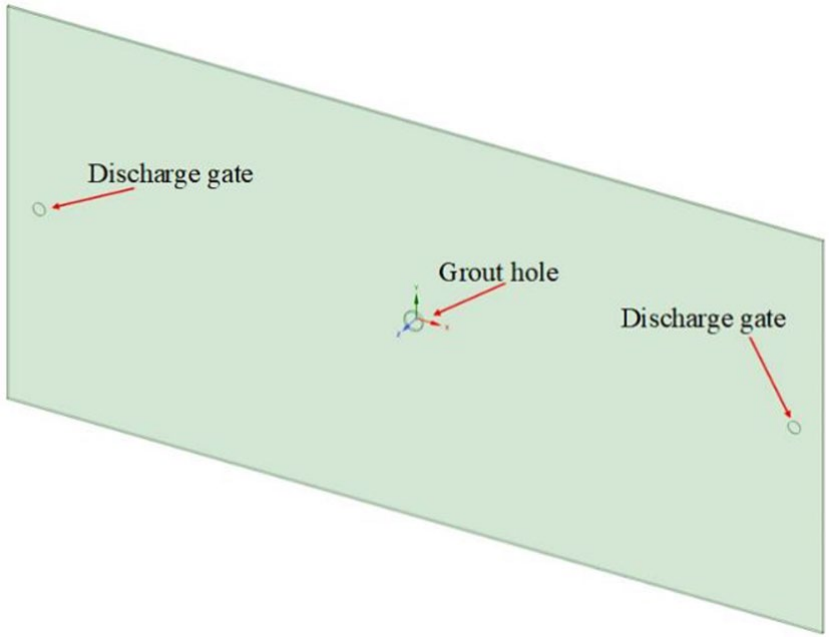
Numerical model of single crack diffusion test.

To solve the above problems, the numerical model of single crack is optimized and improved, from one discharge port on the right to two symmetrical discharge ports on the left and right, to adjust the atmospheric pressure in single crack and make it as close as possible to the laboratory test. The improved single-crack numerical model is shown in Fig. 7.

The improved smooth single crack numerical model is used for grouting simulation calculation. The diffusion time required for each test group and its comparison with the laboratory diffusion test are shown in Table 5.

**Table 5:** Comparison of grouting time between numerical simulation test and laboratory experiment.

Number of groups	1	2	3	4	5
Grouting speed *v*(L/h)	2.1	3.15	4.2	5.25	6.3
laboratory test grouting time *t*(s)	46.2	30.8	23.1	18.4	15.5
Numerical simulation of grouting time *t*(s)	45.2	29.8	22.4	17.8	15
Percentage of grouting time difference	2.2%	4.2%	3%	3.3%	3.2%

**Figure 8:**
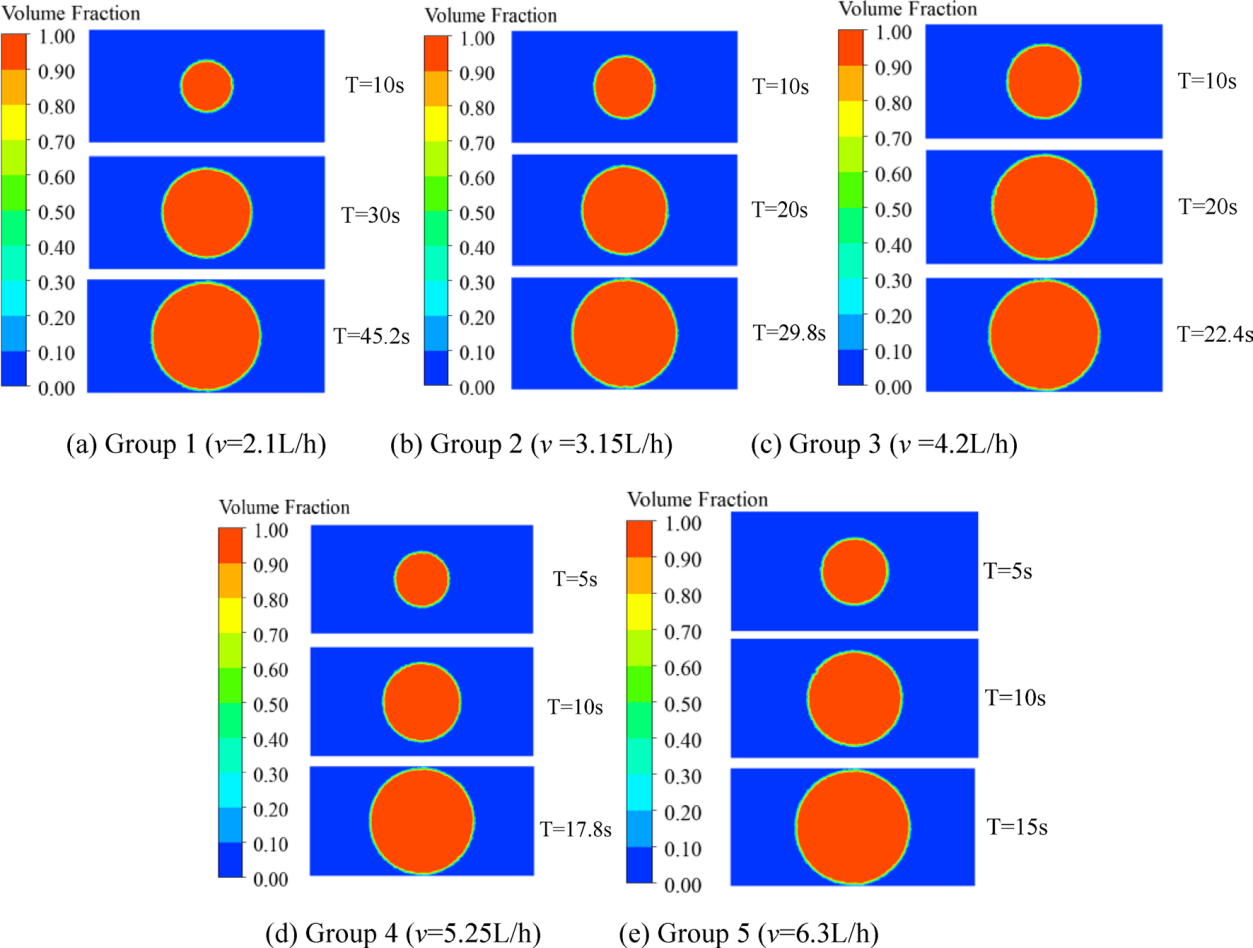
Diffusion cloud map of grouting slurry.

The diffusion cloud of grouting slurry in a smooth single crack at each grouting speed is shown in Fig. 8.

The grouting diffusion time of each test group in numerical simulation and laboratory test are shown in Table 5, and the specific slurry diffusion state is shown in Fig. 8. Compared with the grouting time of the laboratory test, the numerical simulation grouting time of each test group is shorter, but the difference between the grouting time and the laboratory test is not more than 5%, and the consistency is better.

**Figure 9:**
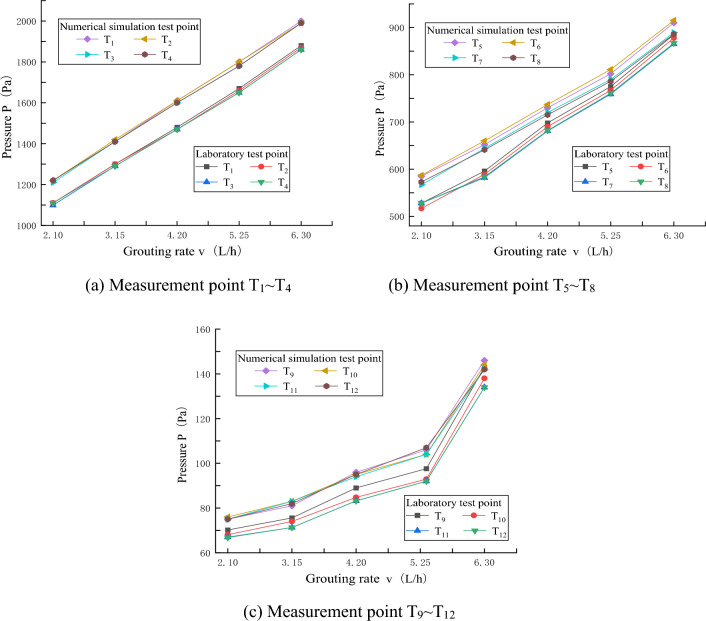
Comparison of pressure curve between laboratory experiment and numerical simulation at different grouting speeds.

The comparison of slurry pressure between the laboratory test and the numerical simulation at each grouting speed is shown in Fig. 9, and it can be obtained that the slurry pressure obtained by the numerical simulation grouting test is larger at the same position from the grouting hole, while the slurry pressure obtained by the laboratory test is smaller. It is believed that the single-crack numerical model can achieve a better sealing effect by setting boundary conditions, resulting in a larger slurry pressure. The slurry pressure value can be obtained by specific analysis, and the slurry pressure of the numerical simulation grouting test is larger, but compared with the pressure value measured in the laboratory grouting test, the difference is less than 10%, which basically meets the test design requirements of numerical simulation. According to the above results, it can be concluded that the numerical model of smooth single crack is correct and can be used to simulate the grouting test of smooth single crack.

## Diffusion law of grouting liquid with different rheological indexes

### Analysis of test scheme

The H-B constitutive model has an important parameter *n*, named as the rheological index (also known as the power law index). Rheological index *n* mainly affects the shear properties of the grout. When *n* > 1, the grout belongs to pseudoplastic fluid and shows shear thickening, namely, the apparent viscosity of the grout increases with the increase of the shear rate. When *n* < 1, the slurry is an expansive fluid, showing shear thinning, namely, the apparent viscosity of the slurry decreases with the increase of shear rate. When *n* = 1, the slurry belongs to Bingham fluid. The diffusion time, diffusion state and pressure field changes of grout with power law coefficient *n* are analyzed.

The crack size is 1350 × 650 × 5mm (length × width × height), and the upper surface of the crack is also provided with 12 measurement points. The distribution of slurry measurement points in the crack model is exactly the same as the laboratory smooth crack grouting test, and the slurry is set to the H-B model. The grouting is carried out at a constant rate, when the slurry diffuses to the crack boundary. When the radial diffusion is near the interface, the grouting is stopped. The numerical simulation of slurry diffusion with regard to the rheological index is shown in Table 6.

**Table 6:** Orthogonal numerical simulation scheme for slurry diffusion (power-law index).

Number of groups	1	2	3	4	5
Grouting speed *v*	4.2	4.2	4.2	4.2	4.2
Roughness *R*	0	0	0	0	0
Crack opening *h*	5	5	5	5	5
Power-law exponent *n*	0.79	0.89	0.99	1.09	1.19
Consistency index *k*	1.51	1.51	1.51	1.51	1.51

### Law analysis of slurry diffusion influence by rheological index

1. The effect of rheological index on the diffusion state of slurry.

After the completion of each group experiment, the slurry diffusion time under different rheological indices is counted, and the resulting slurry diffusion time is shown in Table 7.

**Table 7:** Grouting parameter value (power-law index).

Number of groups	1	2	3	4	5
Grouting speed *v*	4.2	4.2	4.2	4.2	4.2
Roughness *R*	0	0	0	0	0
Crack opening *h*	5	5	5	5	5
Consistency index *k*	1.51	1.51	1.51	1.51	1.51
Rheological index *n*	0.79	0.89	0.99	1.09	1.19
Grouting time *t*	23.1	23	23.2	23.4	23.1

The analysis of the slurry diffusion time of each test group shows that the rheological index has little effect on the diffusion time of the slurry and has little effect on the diffusion state of the slurry.

(2) The effect of rheological index on the pressure field of slurry.

**Figure 10:**
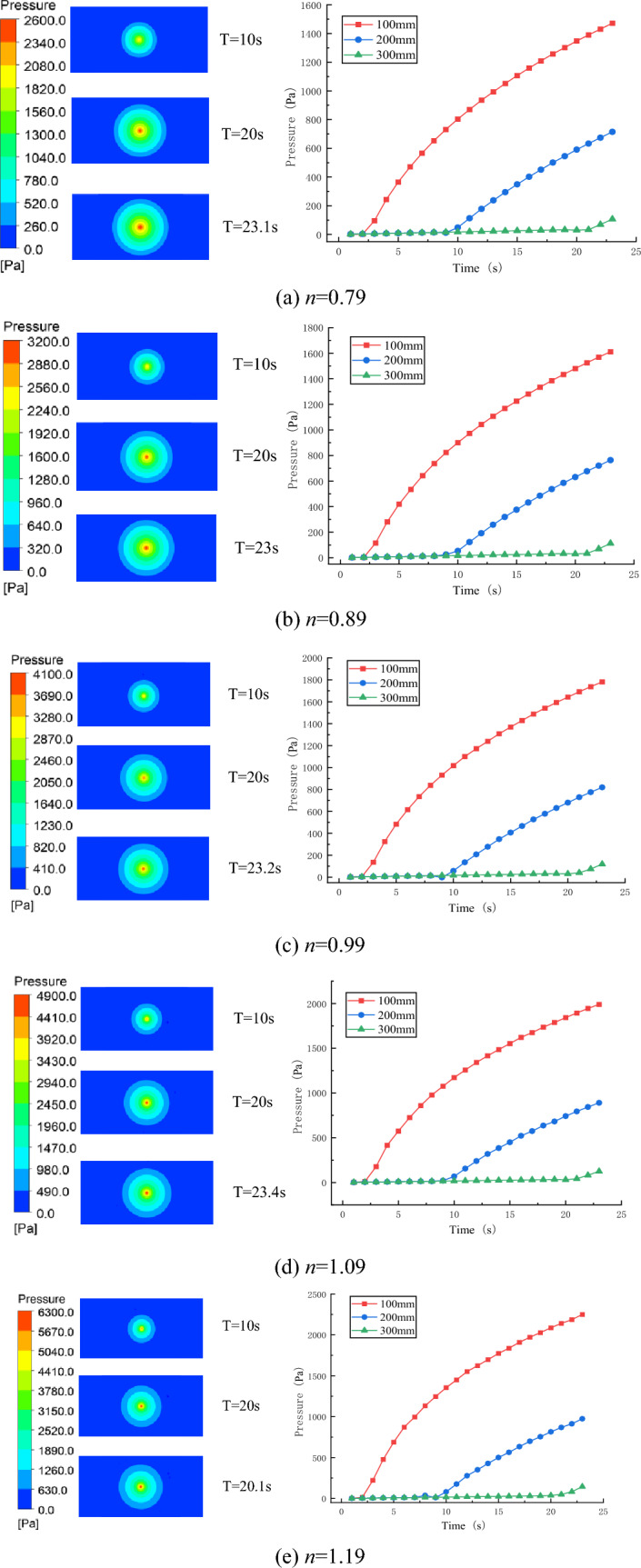
Variation law of slurry pressure field in different rheological indices.

The pressure field distribution of the slurry under each rheological index is shown in Fig. 10.

By analyzing the pressure cloud map of each experimental group, it is obvious that the pressure distribution cloud map of each experimental group has similar rules. The grout pressure value at the grouting hole is the largest and decreases uniformly along the radial direction. When the distance from the grouting hole increases to a certain extent, the grout pressure value basically drops to zero. The closer the distance from the grouting hole is, the larger the grout pressure value is; otherwise, the smaller the grout pressure value is. The grout pressure at the same distance from the grouting hole is basically the same. The grout pressure value at the same measurement point increases with the increase of rheological index, and the grout pressure value at the same distance from the grouting hole is basically the same, which is consistent with the law of pressure field cloud map.

**Figure 11:**
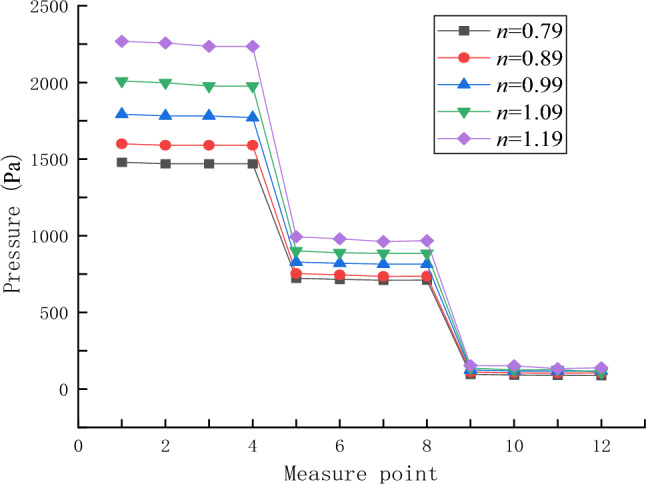
The grout pressure curve of each measuring point under different rheological index.

The pressure curves of 12 measurement points in different rheological indices are shown in Fig. 11. A comparative analysis of the variation curves of grout pressure field in different rheological indices shows that the rheological indices have a great influence on the grout pressure field, and the same law is shown when the distance from the grouting hole is 100 mm, 200 mm and 300 mm, namely, the greater the rheological index is, the greater the grout pressure value is. As constant pressure is used for grouting, concrete analysis shows that the greater the grout pressure value is, the greater the flow resistance of grout is; namely, the greater the rheological coefficient is, the greater the diffusion resistance of grouting slurry is. In engineering practice, when grouting slurry with large rheological index is carried out, the grouting pressure should be increased moderately.

## Diffusion law of grouting slurry with different consistency index

### Analysis of test scheme

Consistency index *k* is also an important parameter in the H-B model, which is a measure of the average viscosity of the fluid, reflecting the viscosity of the slurry. The influence of consistency index *k* on the diffusion time, diffusion state and pressure field of the slurry is analyzed. The crack numerical model, the pressure measurement point distribution and the working conditions are summarized, and the numerical simulation scheme of slurry diffusion on the consistency index is shown in Table 8.

**Table 8:** Orthogonal numerical simulation scheme for slurry diffusion (Consistency index).

Number of groups	1	2	3	4	5
Grouting speed *v*	4.2	4.2	4.2	4.2	4.2
Roughness *R*	0	0	0	0	0
Crack opening *h*	5	5	5	5	5
Power-law exponent *n*	0.79	0.89	0.99	1.09	1.19
Consistency index *k*	1.51	2.51	3.51	4.51	5.51

### Law analysis of slurry diffusion influence by consistency index

The statistical table of grouting time of slurry diffusion test in different consistency indices is shown in Table 9.

**Table 9:** Grouting parameter value (Consistency index).

Number of groups	6	7	8	9	10
Grouting speed *v*	4.2	4.2	4.2	4.2	4.2
Roughness *R*	0	0	0	0	0
Crack opening *h*	5	5	5	5	5
Rheological index *n*	0.79	0.89	0.99	1.09	1.19
Consistency index *k*	1.51	2.51	3.51	4.51	5.51
Grouting time *t*	23	23.2	23.1	22.8	23.2

By analyzing the grout diffusion time of each test group, it is obvious that the consistency index has little influence on the grout diffusion time and the grout diffusion state under the test condition.

**Figure 12:**
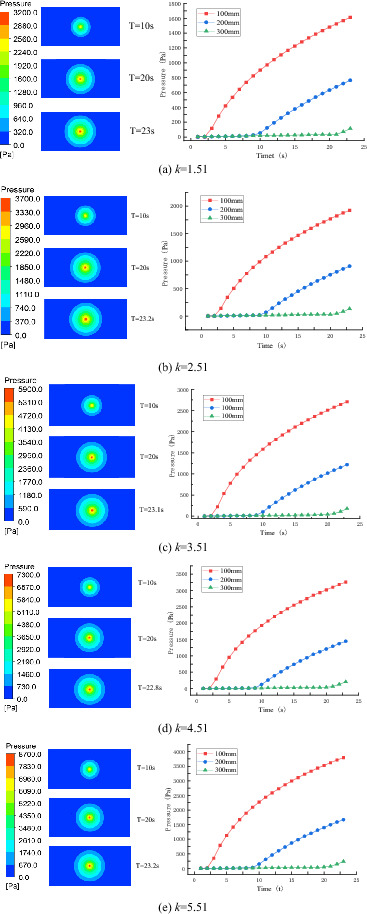
Variation law of slurry pressure field under different consistency index.

The cloud map of slurry diffusion pressure under different consistency indices is shown in Fig. 12. It is obvious that slurry diffusion in different consistency indices still starts from the grouting hole and spreads uniformly around along the radial direction. The pressure value at the same position from the grouting hole is basically the same.

**Figure 13:**
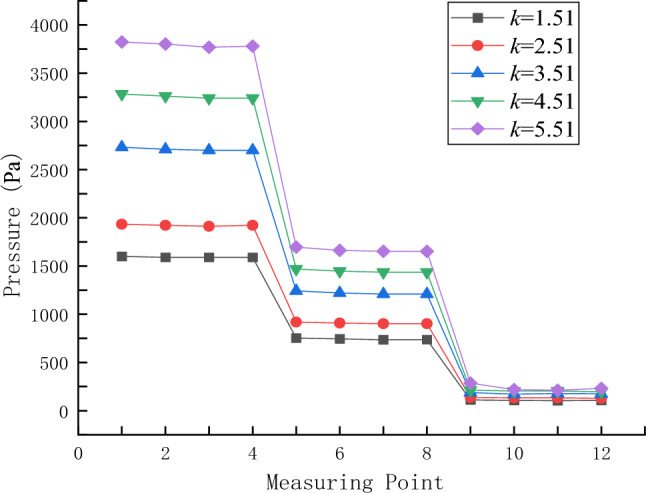
Variation curves of grouting at measurement points in different consistency indices.

Figure 13 is a curve drawn by taking the arithmetic average of the pressure values of four measurement points at the same distance from the grouting hole. The grouting pressure change curves at different consistency indices also show the same trend of change, and the grouting pressure value at each measurement point increases rapidly with the increase of the consistency index.

## Conclusions

1. Basic assumptions of grouting diffusion model of a single slab crack are proposed, and the constitutive equation and diffusion equation of Herschel-Bulkley fluid flow curve are introduced. On this basis, the characteristics and differences of the fluid are analyzed, and the grouting constitutive equation is proposed. The numerical parameters required to simulate slurry diffusion are determined, and a numerical model of grouting diffusion in a single slab crack is built accordingly. The problems of pressure asymmetry and high pressure value at measurement points appear in the numerical simulation, which are analyzed and believed to be caused by the idealized conditions set by the numerical simulation. The same numerical simulation is carried out with the optimized model, and the correctness of the model is verified.
2. The influence of rheological index and consistency index of grouting slurry on the state of grout diffusion is studied. It is found that the grouting slurry uniformly diffuses along the radial direction from the grouting hole in various rheological index and consistency index. At the same distance from the grouting hole, the grout pressure value is basically the same, and the farther from the grouting hole is, the smaller the grout pressure is. In the two kinds of tests, the variation curves of grouting pressure at each measurement point are similar with time, and the grouting pressure increases with the increase of grouting time.
3. The grouting pressure at the same measurement point increases with the increase of rheological index and consistency index. Each 0.1 increase in the rheological index increases the slurry pressure by about 12.5% on the original basis. For every 1 increase in the consistency index, the slurry pressure increases by about 15% on the original basis. It can be concluded that the grouting pressure should be increased moderately when grouting slurry with high rheological index and high consistency index is used in grouting engineering practice.

## Data Availability

Some or all data, models, or codes generated or used during the study are available from the corresponding authors by request.
